# Effect of mild hypothermia preconditioning against low temperature (4°C) induced rat liver cell injury *in vitro*

**DOI:** 10.1371/journal.pone.0176652

**Published:** 2017-04-28

**Authors:** Jiasheng Qin, Yanxing Mai, Yang Li, Zesheng Jiang, Yi Gao

**Affiliations:** 1 Second Department of Hepatobiliary Surgery, Zhujiang Hospital, Southern Medical University, Guangzhou, Guangdong, P.R. China; 2 Institute of Regenerative Medicine, Southern Medical University, Guangzhou, Guangdong, P.R. China; 3 Department of Geriatrics, Zhujiang Hospital, Southern Medical University, Guangzhou, Guangdong, P.R. China; The First Affiliated Hospital of Nanjing Medical University, CHINA

## Abstract

Bioartificial liver holds special position in the field of regenerative medicine, and cold environment at 4℃ is widely used for the short storage of both organ and liver cell for later application. However, the disadvantages of such cold storage could influence cell viability and lead to cell apoptosis in different degrees. In this study, we mainly explore the pre-protective effect of mild hypothermia against low temperature (4℃)-induced rat liver cell injury in vitro. Our results indicated that the precondition with mild hypothermia could increase cell viability, such as cell proliferation, LDH regulation and glycogen synthesis ability of liver cell. The precondition also decreased the ROS production and relieved cell apoptosis in liver cells. Compared with the model group, the mitochondrial membrane potential was restored in the mild hypothermia group, as well as the mitochondrial membrane permeability transition pore opening, indicating that the therapeutic mechanism was related to mitochondrial protection. Further analysis showed that PI3K-Akt-GSK3β signal pathway might be associated with the pre-protective effect of mild hypothermia. Thus, our study suggested that the precondition with mild hypothermia hold the protective effect for liver cell in cold environment, and further developed a novel strategy for the storage of liver seed cells, even bioartificial liver.

## Introduction

With the development of regenerative medicine, bioartificial liver has attracted scientists’ attention in recent years, for its promising application in disease treatment. Somatic liver cells are also widely used for pharmacological and toxicological research, as well as for cell transplantation. In present, cold environment at 2–8℃ (usually at 4℃) is used for the short storage of both the organ and liver cell for later application, and the storage time can vary from several minutes to several hours for different researches. However, scientists have demonstrated the disadvantages of such cold storage could influence cell viability and lead to oxidative damage, mitochondrial dysfunction and cell apoptosis in different degrees [1, 2]. Therefore, the optimized storage method is essential for the application of bioartificial liver and other bioengineering technologies in clinical studies.

Mild hypothermia has been reported to be a very promising neuroprotective therapeutic strategy for patients with brain injury [3–8]. Some groups also demonstrated that the hypothermia could protect liver cell from cell death or apoptosis in some hepatic diseases [9–11]. In recent years, the clinical application of the hypothermia has showed the therapeutic action for the patients with those diseases, and researchers also try to explain the mechanism for the action [12]. For example, mild hypothermia holds the ability to up-regulate the expression of anti-apoptotic gene Bcl-2, and decrease the levels of some inflammatory chemokines (such as IL-8, MCP-1 and COX-2) in endothelial cells [13]. Some scientists also indicated that hypothermia could induce the expression of cold-inducible RNA-binding protein to inhibit cell apoptosis induced by tumor necrosis factor-α via the activation of extracellular signal-regulated kinase [14]. However, most reports describe the complex mechanism about the effect of mild hypothermia on the brain, the detailed molecular mechanisms of underlying potential beneficial effects of hypothermia treatment on the liver cell injury or liver failure may be still far away from our understanding.

As known, cold storage (usually at 4℃) can lead to oxidative damage and cell apoptosis, and liver cell apoptosis is always regarded as an important component of some liver diseases, and the apoptotic signaling pathways mediated by Fas and other apoptotic genes hold significant potential during the process [15–19]. In 2004, Fu et al first evaluated the hepatocyte apoptosis with the treatment of mild hypothermia, and their research indicated that mild hypothermia (26℃) could suppress Fas-mediated apoptotic signaling pathways in liver cells. This function mainly depended on the inhibition of some signaling events, such as cytochrome c release, effector caspase activation, and so on [10]. In recent years, the protective effect of mild hypothermia was also evaluated in some other kinds of liver cell injury models to clarify more detailed mechanisms. Sakurai et al. suggested that hypothermia could protect hepatic cell from cell death through the reduction of ROS production in fulminant hepatitis directly. In their study, concanavalin A-induced hepatitis models were established in mice, and the hypothermia group were kept at 25℃. Their results indicated that hypothermia treatment hold the ability to attenuated liver injury and prolong survival through the activation of c-Jun N-terminal kinase as well as Akt. Similar to their further study about the function of hypothermia in brain injury, the expression of cold-inducible RNA-binding protein was also up-regulated, leading to the decreased hepatocyte apoptosis in the group with mild hypothermia treatment [11, 14].

Therefore, we can conclude that some researchers have demonstrated the protective effect of mild hypothermia from liver cell apoptosis or injury and explored the associated mechanism preliminarily. However, whether the hypothermia still holds the pre-protective effect against liver cell damage and cell apoptisis is still unclear. In this study, low temperature-induced liver cell injury model was established to evaluate the pre-protective effect of mild hypothermia and further establish a novel strategy for the storage of liver seed cells, even bioartificial liver.

## Materials and methods

This study was carried out in strict accordance with the recommendations in the Guide for the Care and Use of Laboratory Animals of the National Institutes of Health. The protocol was approved by the Institutional Animal Care and Use Committee of Southern Medical University.

### Cell culture and treatment

**Fig 1:**
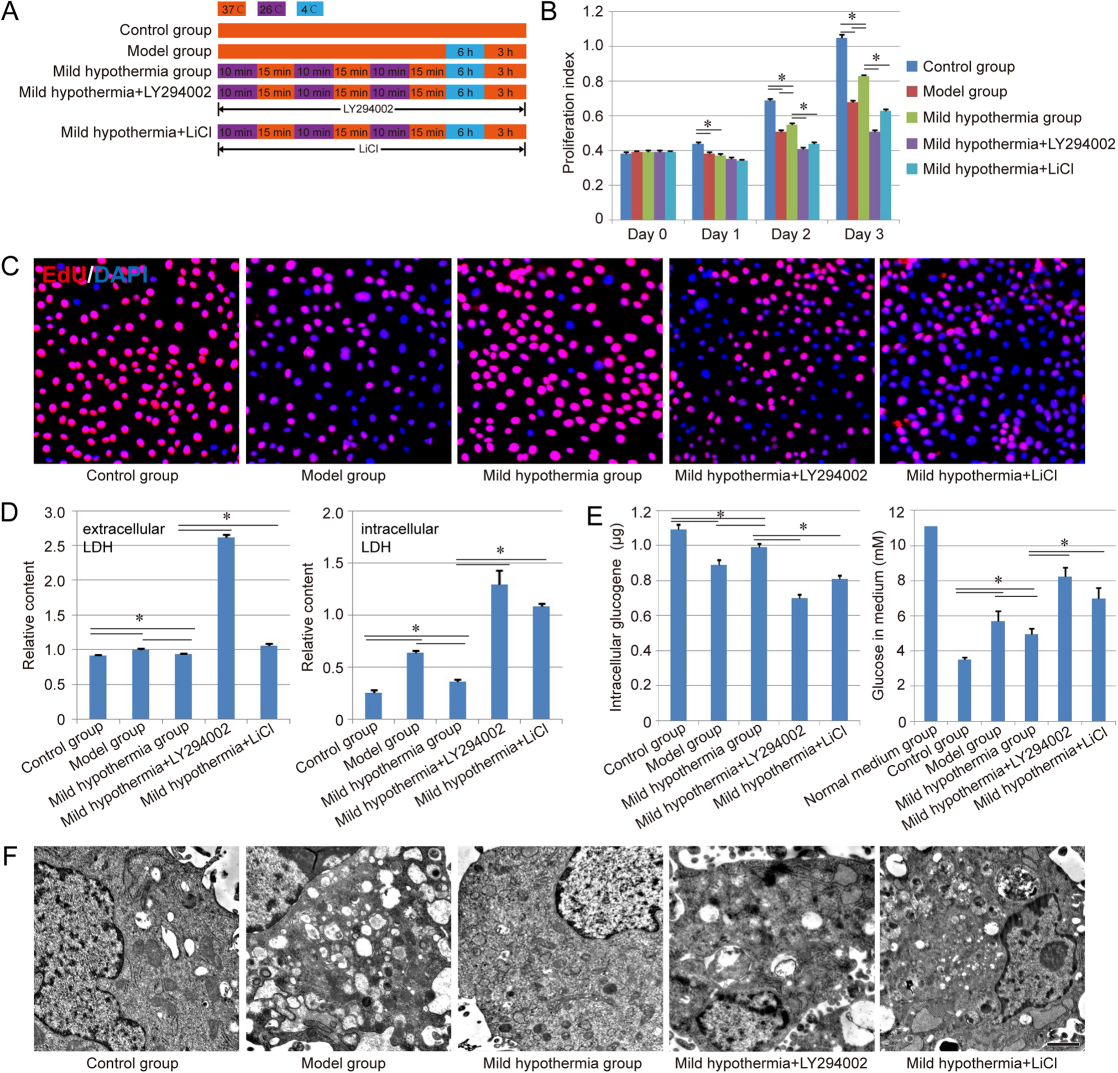
Effect of precondition with mild hypothermia on cell viability. A. Schematic diagram of the approach for treatment of each group. Red orange: the treatment with 37℃; Purple: the treatment with 26℃; Blue: the treatment with 4℃. B-E. Evaluation of cell viability, cell proliferation ability, LDH regulation, glycogen synthesis ability and glucose absorption of each group respectively. Similar results were obtained in three independent experiments and results were expressed as mean ± SEM. A t-test was used to compare the various groups, and *P<0*.*05* was considered statistically significant. *: *P<0*.*05* between the two groups. F. Ultrastructures of Brl-3A cells in each group. Scale bar corresponds to 1 μm.

Rat liver cell line, Brl-3A, was used in our study, which was purchased from the Cell Bank of Type Culture Collection of Chinese Academy of Sciences (Shanghai, China). The cell was cultured in DMEM (Hyclone, glucose concentration = 11.1 mM) supplemented with 10% FBS (Hyclone), 100 U/mL penicillin and 0.1g/mL streptomycin (Hyclone) at 37℃ under 5% CO2 condition. Brl-3A cells cultured in 6-well plate were treated with the hypothermia when the cells were 70–80% confluent, and only 1ml medium was left in each well to ensure the sufficient heat exchange during the treatment process. Five groups were designed as follow (Fig 1A):

1. Control group: Cells were cultured normally in a humidified atmosphere at 37℃ with 5% CO2;
2. Model group: Cells were treated at 4℃for 6 h, and further come back to 37℃ for another 3 h;
3. Mild hypothermia group: Cells were treated with mild hypothermia preconditioning (cells underwent three cycles of incubation at 26℃ for 10min followed with incubation at 37℃ for 15min as the reference [20]), and the following process was the same as the model group;
4. Mild hypothermia+LY294002: All of the operations were the same as the mild hypothermia group. Differently, the medium was further supplemented with 20 μM LY294002 (PI3K inhibitor);
5. Mild hypothermia+LiCl: All of the operations were the same as the mild hypothermia group. Differently, the medium was further supplemented with 20 mM LiCl (GSK3β inhibitor).

### Cell viability assay

Cell viability assay was performed using CCK-8 Kit (Dojindo). Briefly, the cells which were seeded into 96-well plates at a density of 1×10^4^ cells/well, were detected with 10 μL CCK-8, and incubated at 37℃ for 4h. The absorbance was measured at 450 nm. In this study, the proliferation index = the absorbance of experimental group—the absorbance of blank group, was used to evaluate the cell viability of each group. Besides, cell proliferation ability was evaluated with Click-iT® Plus EdU Proliferation Kit (Alexa Fluor® 555, Thermo Fisher Scientific), and the assay was mainly performed according to the manufacturer’s instructions.

To further evaluate cell viability, the extracellular/intracellular LDH, intracellular glycogen and glucose in medium in each group were measured with LDH-Cytotoxicity Colorimetric Assay KitⅡ(BioVision), Glycogen Colorimetric/Fluorometric Assay Kit (BioVision) and Amplex® Red Glucose Assay Kit (BioVision) according to the manufacturer’s instruction respectively.

### Transmission electron microscope (TEM) assay

The cell samples were fixed in glutaraldehyde first, and further dehydrated in graded ethyl alcohols, and embedded with Epon812 finally. In this study, the ultrathin section was cut with an Ultracut E ultramicrotome, and the section was stained with uranyl acetate and lead citrate, and further observed under TEM as previously described [21–23].

### Evaluation of reactive oxygen species (ROS)

To evaluate the oxidative stress in each group, the ROS level of each group was measured using CellROX Orange Reagent (Life Technologies) according to the manufacturer’s instruction. Briefly, Brl-3A cells in each group were collected with trypsin, and washed with PBS for once, then the cells were further incubated with 50 μM CellROX Reagent at 37℃ for 30 min, then washed with PBB for three times. The cells incubated with nothing were used as the negative group. Finally, the cell samples were analyzed using a FACSCalibur cytometer (BD).

### Cell apoptosis assay

In this study, cell apoptosis assay was performed using Vybrant apoptosis Assay Kit (Life Technologies) according to the manufacturer’s instruction. In brief, Brl-3A cells in each group were dissociated into single cells with 0.25% trypsin, and further washed with PBS, then incubated with Hoechst 33342 and PI solution for 30 min. The cells incubated with nothing were used as the negative group. Finally, the cell samples were analyzed using a FACSCalibur cytometer (BD).

### Evaluation of mitochondrial membrane potential (MMP) and mitochondrial membrane permeability transition pore (MPTP)

JC-1 probe (Life Technologies) was used for the evaluation of MMP in each group. After the treatment of each group, the cells were washed with PBS, following by adding 1ml DMEM without FBS, then 1ml JC-1 solution (10 μg/mL) was added, and the system was further incubated at 37℃ for 20 min. The Images of each group were observed under a confocal laser scanning microscopy (Zeiss 710 NLO).

For the detection of MPTP in each group, Living Cell MPTP Fluorescence Detection kit (Genmed) was used in our study, and the operation was mainly performed according to the manufacturer’s instruction. Finally, Samples of all groups were analyzed using a FACSCalibur cytometer (BD).

### Real-Time Quantitative PCR

Total RNA was isolated using Trizol (Invitrogen) according to the manufacturer’s instructions, followed by converting to cDNA using Reverse Transcription System (A3500, Promega). Then, the cDNA samples were used for Real-Time Quantitative PCR (qPCR) with SYBR Green qPCR SuperMix (Invitrogen) on the ABI PRISM 7500 Sequence Detection System. The relative amount of gene transcriptions was normalized to β-actin. The gene expression level of the normal group was regarded as “1.0”, and the relative expression level of the other groups was evaluated. All primer sequences (5’-3’) are as follows:

caspase-3-F: 5’-AATTCAAGGGACGGGTCATG-3’
caspase-3-R: 5’-TGACACAATACACGGGATCTG-3’
caspase-9-F: 5’-CAACAACGTGAACTTCTGCC-3’
caspase-9-R: 5’-GTCAGGTCGTTCTTCACCTC-3’
PI3K -F: 5’-AGTCTGCAGGGACAAAGGAT-3’
PI3K -R: 5’-TGACATGCTGGTTTGAAAGC-3’
AKT -F: 5’-AACCGTGTCCTGCAGAACTC-3’
AKT-R: 5’-CACAATCTCCGCACCGTAGA-3’
GSK-3β-F: 5’- CCACCATCCTTATCCCTCCT-3’
GSK-3β-R: 5’-GTTATTGGTCTGTCCACGGT-3’
β-actin-F: 5’-AGGGAAATCGTGCGTGACAT-3’
β-actin-R: 5’-GAACCGCTCATTGCCGATAG-3’

### Western-blot

The cell samples were harvested with RIPA lysis buffer and the protein content of cell lysates in each group was further determined using BCA protein estimation kit (Pierce, USA). Equal amounts (20 mg) of protein were loaded per lane and electrophoresed in a 10% acrylamide gel (120 V for 1 h). The protein transfer was further performed using nitrocellulose for 1 h at 100 V. The primary antibodies used were anti-Cytochrome (1:500; Santa), anti-PI3K (1:400; Santa), anti-Akt (1:200; Santa), anti-p-Akt (1:200; Santa), anti-GSK-3β(1:200; Santa) and anti-p-GSK-3β (1:300; Santa). Anti-mouse, rabbit or goat HRP and an Amersham ECL kit (GE Healthcare) were further used to detect protein. The band densities were quantified by densitometry (Quantity One v4.62). GAPDH was used for normalization and the relative intensity level of the normal group was regarded as “1.0”, and the relative intensity level of the other groups was evaluated.

### Immunofluorescence staining

The cells were fixed in 4% paraformaldehyde in PBS. For the Immunofluorescence staining, the primary antibodies used were anti-rat voltage dependent anion channel (VDAC; 1:100, Santa), anti-rat hexokinase 2 (HK2, 1:50, Santa). After 12 hours of incubation at 4°C, the samples were washed 3 times with PBS and processed using second antibodies and DAPI, then observed using Axio Scope A1 as the reference [24, 25].

### Statistical analysis

The results were presented as means ± SEM, and the statistical analysis was performed with SPSS16.0. The differences among each group were analyzed by one-way ANOVA and followed by t-test. *P<0*.*05* was considered statistically significant.

## Results

### The pre-protective effect of mild hypothermia in liver cell injury model

The viability of each group was evaluated with CCK-8 method, and the results indicated that the control group hold best potential in cell proliferation compared with other groups. After culture for 2 days, we found that the mild hypothermia group showed better proliferation ability than the model group (*P<0*.*05*), even though still worse than the control group. After adding LY294002 (PI3K inhibitor) or LiCl (GSK3β inhibitor) in the culture system, the viability of mild hypothermia group was further inhibited obviously (*P<0*.*05*), indicating that PI3K-Akt-GSK3β signal pathway might play an important role in the pre-protective effect of mild hypothermia (Fig 1B). Besides, to evaluate the proliferation ability of each group, EdU assay was performed herein. The results indicated that nearly all of cells in the normal group showed positive staining of EdU, while the positive rate was reduced obviously in model group. The mild hypothermia preconditioning could enhance the positive rate significantly, indicating the protection of mild hypothermia from low temperature damage. Similar to CCK-8 result, the treatment of LY294002 or LiCl also could inhibit the pre-protective effect of mild hypothermia, suggesting the significance of PI3K-Akt-GSK3β signal pathway for the pre-protection (Fig 1C).

To further evaluate the cell viability and function, the extracellular/intracellular LDH and intracellular glycogen in each group were measured in our study. Both extracellular LDH and intracellular LDH showed a similar tendency in each group, while the difference of the intracellular LDH among each group was more obvious than that of extracellular LDH. The model group hold higher level of LDH than the control group and mild hypothermia group (*P<0*.*05*), indicating the pre-protective effect of mild hypothermia in the liver cell injury model (Fig 1D). On the other side, capability of glycogenesis was also inhibited in the model group, and the inhibition could be relieved by the precondition with mild hypothermia (*P<0*.*05*). In addition, compared with mild hypothermia group, higher level of LDH and less glycogen could be detected in the groups of mild hypothermia + LY294002 and mild hypothermia + LiCl (*P<0*.*05*, Fig 1D and 1E). We also detected the glucose concentration of each group in medium, and the results indicated that the glucose concentration in each group was down-regulated compared with normal medium group. The mild hypothermia group showed better glucose absorption than the model group (*P<0*.*05*, Fig 1E).

Cell samples from each group were further observed under TEM to evaluate the ultrastructures. The normal Brl-3A cells had clear ultrastructures with minimal heterochromatin and numerous organelles in the cytoplasm. In the model group, lots of vacuoles existed in the cytoplasm, as well as fewer organelles. This damage could be rescued through the precondition with mild hypothermia, and more endocytoplasmic reticulum and mitochondria could be observed in the mild hypothermia group, compared with model group. However, the rescue function also could be disturbed by LY294002 and LiCl in different degree (Fig 1F).

### ROS and cell apoptosis assay

**Fig 2:**
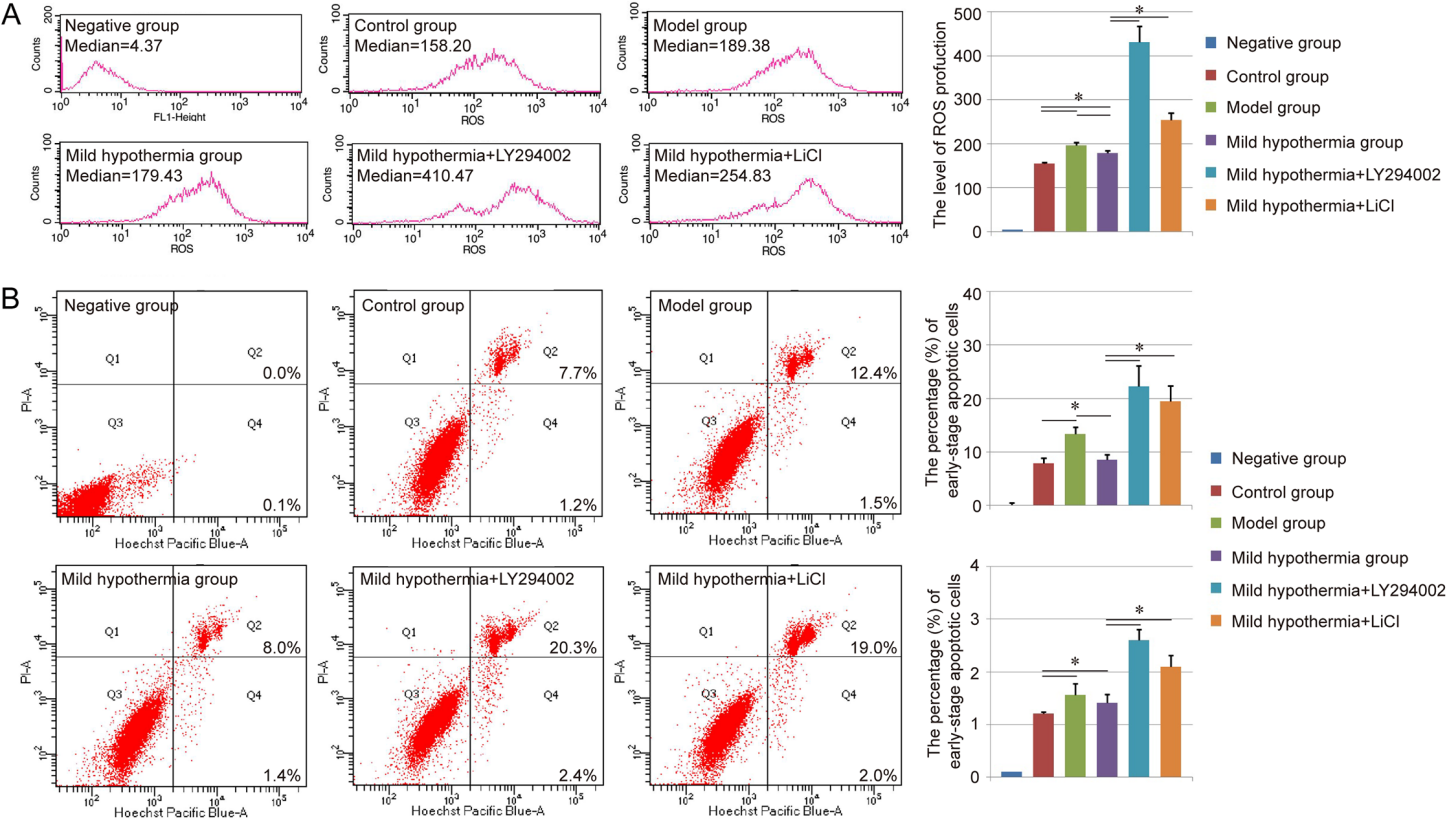
ROS production and Cell apoptosis assay of each group. A. Evaluation of ROS production in each group with FACS. The median of each group was used to evaluate the ROS production. B. Cell apoptosis assay of each group via Hoechst 33342 and PI double staining. Both the early-stage apoptotic cells (Hoechst 33342-positive and PI-negative cells) and late-stage apoptotic cells (Hoechst 33342-positive and PI-positive cells) were analyzed in our study. Similar results were obtained in three independent experiments and results were expressed as mean ± SEM. A t-test was used to compare the various groups, and *P<0*.*05* was considered statistically significant. *: *P<0*.*05* between the two groups.

Scientists have indicated that hypothermia could protect hepatocytes from cell death through the reduction of ROS production in some liver disease [11]. Therefore, the ROS production in each group was evaluated in our study. In the FACS detection, the median of each group was used to evaluate the ROS production. Compared with control group (154.76±2.35), the higher level of ROS could be observed in the model group (196.44±6.57), and a little relief existed in the mild hypothermia group (178.53±6.42). As expected, the treatment of LY294002 and LiCl could further increase the ROS production in the damaged liver cells (Mild hypothermia+LY294002: 430.20±36.76, Mild hypothermia+LiCl: 254.61±15.5, Fig 2A).

Besides, the cell apoptosis of each group was also analyzed through Hoechst 33342 and PI double staining. The staining assay revealed that low temperature-induced damage could lead to an increase in the percentage of early-stage apoptotic cells (Hoechst 33342-positive and PI-negative cells), as well as late-stage apoptotic cells (Hoechst 33342-positive and PI-positive cells) in Brl-3A cells, and further analysis indicated that this apoptosis-induced effect could be inhibited with the precondition with mild hypothermia. Moreover, the groups of mild hypothermia + LY294002 and mild hypothermia + LiCl showed more apoptotic cells in both early stage and late stage than the mild hypothermia group (Fig 2B).

### Evaluation of mitochondrial mechanism

**Fig 3:**
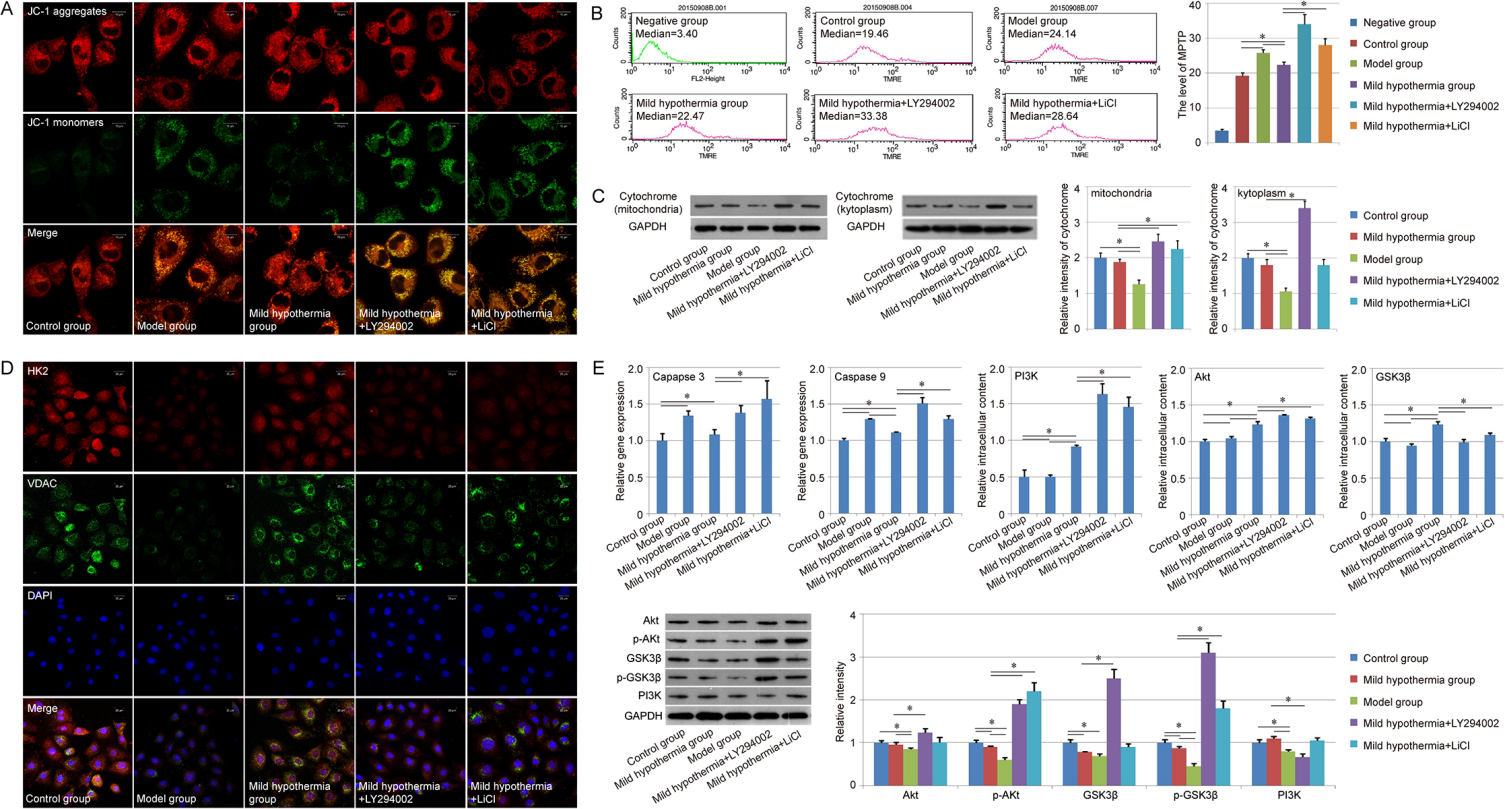
Mitochondrial mechanism of the pre-protective effect of mild hypothermia. A. Evaluation of MMP of each group. Top: Staining of JC-1 aggregates (red); Middle: Staining of JC-1 monomers (green); Bottom: Marge of Top and Middle. Scale bar corresponds to 10 μm. B. Analysis of MPTP opening of each group with FACS. The median of each group was used to evaluate MPTP opening. C. Expression of cytochrome in mitochondria and kytoplasm. D. Evaluation of HK2 (red) and VDAC (green) expression with immunofluorescence. Scale bar corresponds to 20 μm. E. Gene expression assay with qPCR and western-blot. Similar results were obtained in three independent experiments and results were expressed as mean ± SEM. A t-test was used to compare the various groups, and *P<0*.*05* was considered statistically significant. *: *P<0*.*05* between the two groups.

To further analyze the pre-protective effect of mild hypothermia from liver cells damage on the level of subcellular organelle, both MMP and MPTP were analyzed in each group. Herein, JC-1 staining assay was carried out to determine the alteration of MMP. All of groups showed positive staining of JC-1 aggregates (red), while the staining of JC-1 monomers (green) was different in each group. Few JC-1 monomers could be observed in the control group, and the amount of JC-1 monomers was increased in the damaged liver cells. The mild hypothermia also exhibited the pre-protective effect and the number of cells with loss of mitochondrial membrane potential was decreased with the precondition (Fig 3A). Similar results were achieved in the MPTP detection with FACS, in which the median was also used to evaluate the MPTP in each group. Compared with control group (19.20±0.93), the MPTP of the model group (25.80±1.21) was increased in some degree. A little relief could be observed in the mild hypothermia group (22.41±0.78) (Fig 3B). In addition, LY294002 and LiCl could promote the loss of mitochondrial membrane potential in the detection of MMP and MPTP (Fig 3A and 3B). The cytochrome in mitochondria and kytoplasm was further detected respectively. We found that the amount of cytochrome in mitochondria and kytoplasm was reduced in the model group, compared with the control group and mild hypothermia group. However, amount of cytochrome in mitochondria and kytoplasm didn’t show obvious difference between the control group and mild hypothermia group (Fig 3C). Besides, the expression of HK2 and VDAC, two kinds of mitochondrial membrane proteins, were evaluated with immunofluorescence in each group, and the results showed the down-regulation of HK2 and VDAC expression in the model group and up-regulation in the mild hypothermia group, indicating the relationship between the pre-protective effect of mild hypothermia and the regulation of mitochondrial membrane protein expression (Fig 3D).

We also detected the expression of Caspase 9, a gene associated with mitochondrial apoptotic pathway. The results indicated that the expression of Caspase 9 was up-regulated in the model group compared with the control group, and further down-regulated in the mild hypothermia group (*P<0*.*05*, Fig 3E). Similar results could be found in the expression of Caspase 3, another apoptotic gene, in each group (Fig 3E). With the treatment of LY294002 and LiCl, expression of the two apoptotic genes could be up-regulated obviously, indicating the function of PI3K-Akt-GSK3β signal pathway in the pre-protective effect of mild hypothermia (Fig 3E). Therefore, we further detected the expression of PI3K, Akt, and GSK3β in each group. The qPCR results suggested that the precondition with mild hypothermia could increase the expression of those genes compared with model group (*P<0*.*05*) (Fig 3E). In the level of protein, modest up-regulation of the three genes expression could be observed in the mild hypothermia group compared with model group. In addition, we also detected the functional proteins in PI3K-Akt-GSK3β signal pathway, p-Akt, and p-GSK3β, and the result showed that the expression of p-Akt and p-GSK3β were decreased in the model group obviously, and the precondition with mild hypothermia also could increase the expression of p-Akt and p-GSK3β, suggesting that the pre-protective effect of mild hypothermia depended on the function of PI3K-Akt-GSK3β signal pathway in some degree (Fig 3E). With the treatment of inhibitors, qPCR results showed that the expression of GSK3β was down-regulated in the group of mild hypothermia + LY294002 and mild hypothermia + LiCl, while the expression levels of PI3K and Akt were up-regulated in those groups. Western-blot results also indicated the up-regulation of p-Akt and p-GSK3β in those groups (Fig 3E). This phenomenon might be due to the negative feedback control mechanisms in the culture system.

## Discussion

Although several groups have indicated that mild hypothermia had a protective effect against liver cell injury, the pre-protective effect of hypothermia on the liver cell has not been determined so far [9–11]. In the present study, our results indicated that the precondition with mild hypothermia could inhibit ROS production and cell apoptosis and increase cell viability of Brl-3A cell line, and develop a novel strategy for the storage of liver cell. Scientists have demonstrated that ROS are considered as a major mediator of inflammation and the reduction of ROS levels can lead to the attenuation of liver cell injury and apoptosis directly [26–30]. Therefore, the mild hypothermia group holds better ability on cell proliferation, LDH regulation and glycogen synthesis. However, Brl-3A cell line used in our study is different from primary hepatocype, which are always applied in clinic. For example, the Brl-3A cells hold strong proliferative ability and stable hepatic function, while primary hepatocype could only maintain their hepatic function and proliferative ability for a short period in vitro. Even though Brl-3A cell line is regarded as a good hepatic cell model and used to evaluate the liver cell function in vitro widely [31–34], the exploration with primary hepatocyte should hold more clinical significance. Our group isolated rat primary hepatocyte as the reference [35] and further analyzed the effect of hypothermia on cell viability and cell proliferation ability. Similar to Brl-3A cell line, our results indicated that precondition with mild hypothermia still hold protective function on rat primary hepatocyte (S1 Fig). However, the detailed mechanism about such phenomenon still need our further investigation.

Besides, we detected the temperature changing during the hypothermia preconditioning process (S2 Fig), and the result also indicated that 10 min or 15 min was enough for the heat exchange in our culture system. However, more than 10^9^ cells are needed for bioartificial liver normally [36–38]. Both the cell number and medium are much more than that used in our study. Therefore, the either 10min or 15min treatment indeed is not suitable for such large-scale liver cell storage or real bioartificial liver. Therefore, the accurate time of mild hypothermia preconditioning still needs further preclinical exploration, even clinical study.

In addition, some scientists also indicated that the therapeutic mechanism of mild hypothermia may be related to mitochondrial protection. Mild hypothermia treatment could decrease MPTP opening and restore the MMP. Furthermore, hypothermia also could decrease mitochondrial malondialdehyde and elevate mitochondrial-reduced glutathione, consistent with the restored mitochondrial function [39]. However, the mechanisms about the reduction in MMP by mild hypothermia are complex [40]. For example, ischemia-reperfusion could increase the level of MPTP activators (Ca2þ, Pi and ROS,) and reduce the level of MPTP inhibitors (ATP/ADP and low PH) [41]. Both the activator and inhibitors of MPTP opening could be impacted by the mild hypothermia in different degree, and the balance between the activator and inhibitors determined the duration of MPTP opening [39, 40, 42]. In this study, the reduction of MPTP opening and the restoration of MMP were also observed in the mild hypothermia group, as well as the inhibition of ROS production, a MPTP activator. The expression of Caspase 9, a gene associated with mitochondrial apoptotic pathway, was also down-regulated in the mild hypothermia group, compared with model group. Further detection showed that the expression of both HK2 and VDAC, two kinds of membrane proteins of mitochondria, could be down-regulated with low temperature treatment, and mild hypothermia preconditioning could up-regulated their expression in damaged hepatic cells. Thus, the pre-protective effect of mild hypothermia should also be related to mitochondrial protection, and HK2 and VDAC might be the target proteins during the protection process. However, whether the detailed mechanism of MMP and MPTP regulation is similar to the previous reports, still needs our further exploration.

In our research, we found that the effect of precondition with mild hypothermia could be inhibited through adding LY294002 (PI3K inhibitor) or LiCl (GSK3β inhibitor) in the culture system. The results suggested that PI3K-Akt-GSK3β signal pathway might play an important role in the pre-protective effect of mild hypothermia. PI3K-Akt-GSK3β signal pathway has been demonstrated to be essential for the recovery of liver cell injury by some groups [43–47]. The pathway also has been indicated to hold the ability to regulate the protective effect of mild hypothermia against ischaemia/reperfusion injury. The expression levels of p-Akt, and p-GSK3β in the mild hypothermia treated group were higher than those in the ischaemia/reperfusion group, and the therapeutic function also could be influenced by LY294002 [48, 49]. In our study, the results showed that the cell viability, MMP and other characteristics were much worse in the group of mild hypothermia + LY294002 and mild hypothermia + LiCl than those in the control group, the mild hypothermia group, even the model group, indicating that the PI3K-Akt-GSK3β signal pathway might be associated with not only pre-protective effect of mild hypothermia, but also other essential functions in liver cell. Interestingly, with the treatment of LY294002 and LiCl, the expression levels of some key genes in the pathway were up-regulated in those groups. Maybe this phenomenon was due to the negative feedback control mechanisms in the low temperature-induced liver cell injury model. Anyway, this conclusion still needs our further confirmation. In addition, our study only evaluated the expression of PI3K, Akt/p-Akt and GSK3β/p-GSK3β in the signal pathway with qPCR and western-blot preliminary, more detailed and accurate analysis about the pathway, such as the expression of upstream or downstream genes, should also be investigated intensively.

## Conclusion

In conclusion, this study mainly investigated the pre-protective effect of mild hypothermia on low temperature-induced liver cell injury, and provided a novel strategy for the storage of liver cell in bioengineering. The results indicated that the precondition with mild hypothermia could enhance cell proliferation, LDH regulation and glycogen synthesis ability, and relief the ROS production and cell apoptosis in Brl-3A cells. Further analysis showed that the precondition also could decrease MPTP opening and restore MMP, and PI3K-Akt-GSK3β signal pathway might be associated with the pre-protective effect of mild hypothermia. However, the detailed mechanism of this effect still needs our further exploration.

## Supporting information

S1 Fig Effect of precondition with mild hypothermia on rat primary hepatocyte. A. Evaluation of cell viability with CCK-8. B. Analysis of cell proliferation ability with EDU staining. Similar results were obtained in three independent experiments and results were expressed as mean ± SEM. A t-test was used to compare the various groups, and *P<0*.*05* was considered statistically significant. *: *P<0*.*05* between the two groups.
(PDF)

S2 Fig The temperature changing during the hypothermia preconditioning process. (PDF)
