# Supplementary figures and images for: Effect of mild hypothermia preconditioning against low temperature (4°C) induced rat liver cell injury in vitro
Source: PLoS One. 2017 Apr 28;12(4):e0176652. doi: 10.1371/journal.pone.0176652 (PMC5409157; doi:10.1371/journal.pone.0176652)

# Supplementary Figure 1

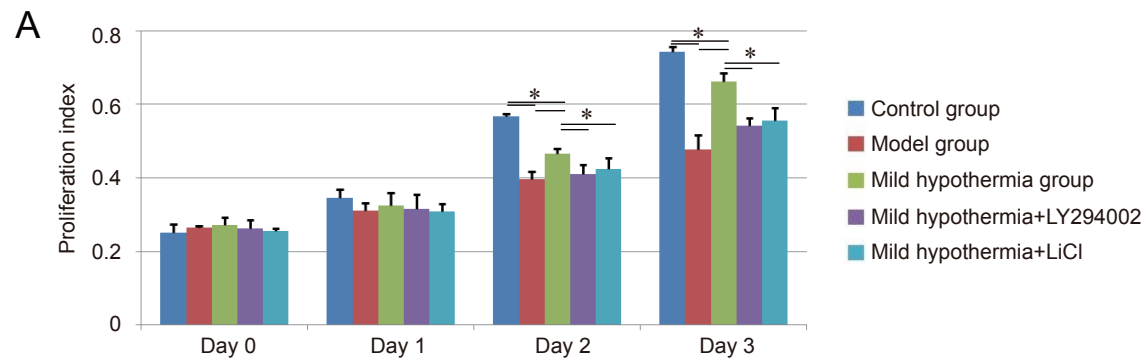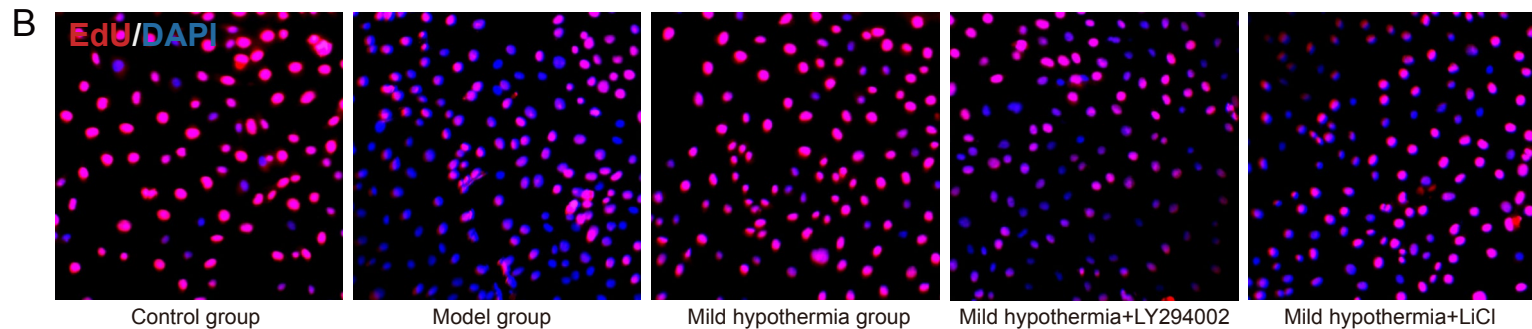

Supplement: S1 Fig — A. Evaluation of cell viability with CCK-8. B. Analysis of cell proliferation ability with EDU staining. Similar results were obtained in three independent experiments and results were expressed as mean ± SEM. A t-test was used to compare the various groups, and P<0.05 was considered statistically significant. *: P<0.05 between the two groups. (PDF) [file pone.0176652.s001.pdf]

Supplementary Figure 2

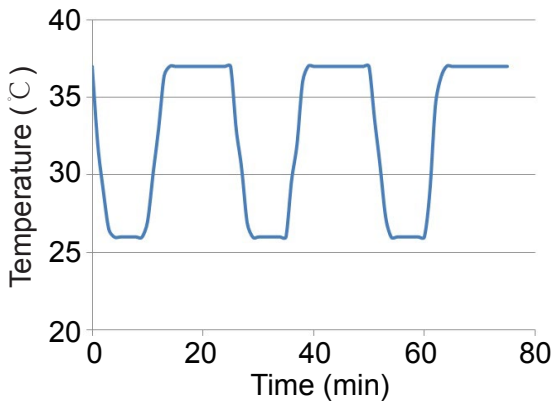

Supplement: S2 Fig — (PDF) [file pone.0176652.s002.pdf]
